# Antisemitism as a Chronic Cardiometabolic, Endocrine, and Glutamatergic Stressor Requiring Psychometric Monitoring

**DOI:** 10.1055/a-2869-4941

**Published:** 2026-05-29

**Authors:** Barbara Ettrich, Ulrike M. E. Schulze, Jochen Hardt, Oliver Decker, Stefan R. Bornstein, Enrico Ullmann

**Affiliations:** 1 PSYCHONEUROLOGIE des LEBENSWEGEnSLeipzigGermany; 2 Department of Child and Adolescent Psychiatry, Psychosomatics and Psychotherapy 27197 Ulm University HospitalUlmBWGermany; 3 Department of Child and Adolescent PsychiatryCenter for Psychiatry CalwBöblingenGermany; 4 Department of Medical Psychology and Sociology 39068 Johannes Gutenberg University Hospital MainzMainzRPGermany; 5 Department of Medical Psychology and Medical Sociology 70622 University of Leipzig Faculty of MedicineLeipzigSNGermany; 6 Else Frenkel Brunswick Institute 9180 Leipzig UniversityLeipzigSNGermany; 7 Faculty of Life Sciences and Medicine 4616 King’s College LondonLondonUnited Kingdom of Great Britain and Northern Ireland; 8 Department of Medicine III 39063 Universitatsklinikum Carl Gustav CarusDresdenGermany

**Keywords:** antisemitism, chronic stress, pituitary, HPA axis, glutamate, allostatic load, cardiometabolic risk

## Abstract

We are seeing an exploding expansion of antisemitic attacks worldwide, raising concerns about their potential impact on biological stress regulation and health. Antisemitism is a historically persistent and structurally embedded form of social exclusion that may contribute to chronic psychosocial stress exposure. Building on research into intergenerational trauma, including neuroendocrine alterations in Holocaust survivors and their descendants, this commentary integrates psychometric, empirical, and conceptual approaches to propose a biologically grounded framework linking antisemitism to endocrine and cardiometabolic processes. Preliminary findings from a pilot study using a Checklist of Antisemitic Perception instrument, in combination with established psychometric measures, indicate an increased psychological burden associated with antisemitic experiences, with clinically relevant symptom levels observed across groups. Mechanistically, chronic stress is mediated by neuroendocrine pathways involving the hypothalamic–pituitary–adrenal axis, autonomic nervous system, and immune regulation, contributing to allostatic load and cardiometabolic risk. Emerging evidence suggests that stress responses are heterogeneous and influenced by individual coping styles, with distinct allostatic set-points and associated neurobiological adaptations, including alterations in striatal glutamatergic signaling. Institutional and discursive contexts may further modulate exposure to antisemitic stressors, as reflected in heterogeneous professional engagement and variations in thematic emphasis within medical discourse. Taken together, these observations support the conceptualization of antisemitism as a chronic stressor with potential biological consequences and highlight the importance of integrating psychometric and biological approaches in future research.

## Introduction

Social conditions are increasingly recognized as key determinants of health, with chronic psychosocial stress emerging as a central factor linking social environments to endocrine and metabolic diseases. Since Durkheim’s early work, it has been established that social integration and exclusion are associated with measurable differences in morbidity and mortality, underscoring the biological relevance of societal structures. 1​ This perspective is closely aligned with classical concepts in social medicine articulated by Rudolf Virchow, who emphasized that social and economic conditions fundamentally shape disease patterns and health outcomes. 2​

Over the past decade, and particularly following the terrorist attacks of October 7, 2023, a marked increase in antisemitic incidents has been observed worldwide. In the United States, the largest Jewish community outside Israel, reported antisemitic assaults increased from 3,697 in 2022 to 7,523 in 2023. 3​ ​4​ Similarly, in France, reported incidents rose from 436 to 1,667 within the same period. In Germany, antisemitic crimes—including verbal and physical acts—rose by approximately 95% in 2023 compared to the previous year, reaching the highest level since World War II, with more than 4,500 documented incidents despite a Jewish population representing less than 1% of the total population. 5​ ​6​ These figures likely underestimate the true burden due to under-reporting.

While discrimination affects multiple minority groups and may differ in form and prevalence, recent developments underscore the magnitude and persistence of antisemitism as a contemporary social stressor. Although other forms of group-directed hostility, including Islamophobia, represent important and distinct expressions of social exclusion, available data suggest that antisemitism currently exhibits particularly high incidence rates in several Western countries, underscoring its relevance as a psychosocial stressor. 7​

The term “antisemitism” itself has been described as a historically constructed and, in part, pseudo-scientific concept originating in the 19 ^th^ century, despite the broader linguistic meaning of “semitic” referring to multiple populations. In its historical and contemporary usage, however, the term has been specifically applied to hostility directed against Jews, reflecting a distinct form of prejudice embedded in European intellectual and political history.

Antisemitism represents a specific form of discrimination characterized by features that distinguish it from other forms of social exclusion, without implying a hierarchy of suffering. Rather than being limited to visible or socio-demographic markers, antisemitism is frequently embedded in enduring ideological narratives that persist across centuries and across changing political contexts. This distinctiveness has been described in historical and sociological research emphasizing the persistence, adaptability, and ideologically embedded nature of antisemitism across different societal contexts. 7​

Early clinical observations by Niederland described the “survivor syndrome” in Holocaust survivors, highlighting the long-term psychological and somatic sequelae of extreme trauma. 8​ Subsequent neuroendocrine studies by Yehuda and colleagues demonstrated alterations in cortisol regulation in Holocaust survivors and their offspring, including evidence of altered glucocorticoid (GR) sensitivity and epigenetic modifications such as FKBP5 methylation. 9​ 10​ ​11​ These findings support the concept of intergenerational adaptation of stress-response systems. Extending this line of research, our previous work and related studies in the third-generation offspring of Holocaust survivors demonstrated elevated cortisol levels associated with antisemitic experiences and specific parental rearing patterns, suggesting that contemporary social stressors continue to shape neuroendocrine functions. 12​

In parallel, a growing body of recent research has begun to examine the psychosocial and health-related consequences of antisemitism in contemporary contexts. Studies conducted in the aftermath of October 7, 2023 have documented increased psychological distress, anxiety, and perceived threat among Jewish populations, particularly in academic environments such as university campuses. Furthermore, recent investigations in European settings have highlighted the broader psychosocial burden associated with antisemitic experiences, while emerging work suggests potential links between sustained exposure to antisemitism and cardiometabolic risk. These findings underscore the increasing relevance of systematic tools to assess antisemitism-related stress exposure and support the need for integrative approaches combining psychometric and biological perspectives. 13​

Despite these advances, systematic tools to assess contemporary antisemitism-related stress exposure remain limited. This gap is particularly relevant given the increasing need to quantify psychosocial stressors in both research and clinical contexts. To address this gap, the Checklist of Antisemitic Perceptions (CLASP; Table 1 ) instrument was developed as a psychometric tool capturing contemporary antisemitic experiences and their psychological correlates.

**Table:** **Table 1** The CLASP instrument assesses direct and indirect exposure to antisemitic experiences, including verbal, ideological, social, and physical forms of antisemitism. Items capture personally experienced, witnessed, and indirectly learned events, as well as perceived antisemitic attitudes and stress-related perceptions. Responses are rated on a five-point scale ranging from direct personal exposure (“happened to me”) to non-applicability (“does not apply”).

Event		(i) Happened to me	(ii) Witnessed it	(iii) Learned about it	(iv) Not sure	(v) Does not apply
**a**	Jewish people and families like George Soros and the Rothschilds have too much influence in the world and the media.	4	3	2	1	0
**b**	Jewish people are cunning and more dishonest than non-Jewish people to achieve their goals.	4	3	2	1	0
**c**	The Central Council of Jews in Germany should be abolished because it disrupts social peace in Germany.	4	3	2	1	0
**d**	The Jews think that they are a chosen people above other people.	4	3	2	1	0
**e**	Demands for reparations often no longer benefit the victims, but rather a Holocaust industry of resourceful lawyers.	4	3	2	1	0
**f**	If you look for anti-semitism, you will find it. Jewish people must free themselves from the victim mentality.	4	3	2	1	0
**g**	Legitimate criticism of Israel is deliberately silenced by accusations of anti-semitism.	4	3	2	1	0
**h**	Jewish people only talk about the Holocaust to advance their personal agenda.	4	3	2	1	0
**i**	Jews are so sensitive. Better do not discuss sensitive topics with Jewish people because you are often misunderstood and have the feeling that words are being put in your mouth even though you did not mean them.	4	3	2	1	0
**j**	Israeli politics are making Jewish people increasingly unsympathetic.	4	3	2	1	0
**k**	What the State of Israel is doing to the Palestinian people today is essentially the same as what the Nazis did to Jewish people in the Third Reich.	4	3	2	1	0
**l**	Just because it is written somewhere in the Bible does not mean that Jews in Israel should be allowed to have their own state.	4	3	2	1	0
**m**	In a democracy, it should also be possible to critically question the Holocaust.	4	3	2	1	0
**n**	Israel is waging a war of annihilation in the territories of Palestine and committing genocide as in apartheid.	4	3	2	1	0
**o**	Today, we should stop talking so much about the Holocaust and finally draw a line in the sand.	4	3	2	1	0
**p**	It was a wrong decision to give the Jews a state in Palestine because the conflict was predestined.	4	3	2	1	0
**q**	Assault with a weapon (for example, being shot, stabbed, threatened with a knife, gun, and bomb)	4	3	2	1	0
**r**	Sexual assault (rape, attempted rape, and forced to perform any type of sexual act by force or threat of force)	4	3	2	1	0
**s**	Captivity (e.g., kidnapped, taken hostage, and a prisoner of war)	4	3	2	1	0
**t**	Combat operations or stay in a war zone (military or civilian).	4	3	2	1	0
**u**	Other unwanted or unpleasant sexual experiences	4	3	4	1	0
**v**	Physical assault (for example, being attacked, hit, slapped, kicked, and beaten up)	4	3	2	1	0
**w**	Damage of ownership (car, house, apartment, and property)	4	3	2	1	0
	Any other very stressful event or experience related to an anti-semitic background.					
	x: ___________________________________	4	3	2	1	0
	y: ___________________________________	4	3	2	1	0
	If you experienced more than one of the events asked, which was the worst/most distressing?					
	Event letter:					

Instructions: Listed below are a number of difficult or stressful verbal and physical perceptions that sometimes happen to people. For each event check one or more of the boxes to the right to indicate that: (i) it happened to you verbal/physical; (ii) you witnessed it verbal/physical to someone else; (iii) you learned about it verbal/physical to a close family member or close friend; (iv) you’re not sure if it fits; or (v) it doesn’t apply to you. Be sure to consider your entire life (growing up as well as adulthood) as you go through the list of events.

Beyond these specific contexts, a substantial body of research has demonstrated that discrimination constitutes a chronic psychosocial stressor with measurable effects on both mental and physical health. Meta-analytic evidence indicates that perceived discrimination is associated with heightened physiological stress responses, including increased cardiovascular reactivity and cortisol secretion, as well as long-term processes of allostatic load that contribute to cardiometabolic risks. These effects are particularly pronounced when stressors are experienced as uncontrollable and unpredictable—features that are characteristic of many forms of discrimination. 14​

Within this broader framework, institutional environments represent an important contextual layer that can modulate the experience and impact of psychosocial stressors.

In Germany, a few State Chambers of Physicians have taken explicit steps to address antisemitism, including engagement with the association “Ärztinnen und Ärzte gegen Antisemitismus,” while others have not pursued comparable forms of engagement, frequently citing institutional neutrality or broader policy considerations (personal communications from State Chambers of Physicians, January-March 2026). Of 17 State Chambers of Physicians contacted, 2 formally supported membership initiatives, 9 declined, and 5 did not respond. At the same time, the 130th German Medical Assembly (2026) adopted and referred to the executive board a proposal supporting the establishment of a sensitive nationwide contact structure on antisemitism within the German Medical Association. From a psychosocial perspective, such institutional differences may be relevant because organizational environments can influence perceived recognition, social support, and psychological safety, factors known to modulate stress perception and coping processes. Similar variations in thematic emphasis have also been observed within scientific discourse, including a quantitative imbalance in Gaza- and Israel-related publications in ​​​​ *The Lancet* , despite existing commitments such as the Holocaust Commission emphasizing balanced and responsible engagement with antisemitism. 15​ 16​ ​17​

The present commentary integrates three complementary components. First, we introduce the CLASP as a psychometric instrument designed to capture contemporary antisemitic stress exposure. Second, we present preliminary pilot data illustrating the application of this instrument and its association with psychological outcomes. Third, we outline a biologically grounded conceptual framework linking chronic antisemitism-related stress to neuroendocrine, metabolic, and neurobiological processes. Together, these elements aim to provide a structured foundation for future hypothesis-driven research at the intersection of social stress, biology, and health.

Taken together, these observations suggest that antisemitism may function as a chronic psychosocial stressor with potential consequences for neuroendocrine regulation and cardiometabolic health.

## Preliminary findings

To complement this conceptual framework, preliminary empirical observations provide initial evidence supporting the proposed relationship between antisemitic stress exposure and health-related outcomes.

A pseudonymized online survey was conducted in November 2024, including 28 Jewish and non-Jewish participants recruited through two civil society organizations engaged in addressing antisemitism (“Ärztinnen und Ärzte gegen Antisemitismus e. V. *”* and “Netzwerk jüdischer Hochschullehrender e.V. *”* ). The inclusion of both groups enabled exploratory comparisons of stress-related outcomes. The study was designed as a pilot and was not intended to yield representative estimates.

Antisemitic experiences were assessed using the CLASP instrument, capturing both direct and indirect exposure, including perceived discrimination, social exclusion, and anticipatory stress. These measures were combined with established psychometric instruments assessing anxiety (GAD-7), depressive symptoms (PHQ-9), somatic complaints (SSS-8), and post-traumatic stress (PCL).

The results ( Table 2 ) indicated a consistently higher burden of depressive, anxiety, somatic, and post-traumatic stress symptoms in Jewish participants compared to non-Jewish participants. Although differences in composite scores did not reach statistical significance, the directionality of effects was uniform across measures, suggesting a coherent pattern of increased psychological burden. Importantly, clinically relevant symptom levels were observed in both groups. Five participants met criteria consistent with post-traumatic stress disorder, including two non-Jewish and three Jewish individuals. This finding indicates that antisemitism-related stress exposure may extend beyond directly targeted individuals and influence broader psychosocial environments.

**Table:** **Table 2** Mental burden in subjects with and without Jewish background

	Jewish subjects	Non-Jewish subjects
Sample size	14	14
Mean age (y)	53.3	51.8
Age range (y)	36–70	21–79
**1. Self-report scales**		
	Mean±SD	Mean±SD
GAD-7 (anxiety)	8.4±7.38	5.4±3.50
PHQ-9 (depressiveness)	6.1±5.85	4.6±4.20
SSS-8 (somatic burden)	8.5±7.16	7.1±5.82
PCL whole	20.43±19.44	6.8±7.15
**2. Symptom cluster of PTSD**		
	Sum	Sum
PCL B-criteria	84	32
PCL C-criteria	18	5
PCL D-criteria	54	36
PCL E-criteria	76	22
**3. Total burden of complaints**		
Two-Sample two-sided *T* -test: 10.9±12.68 (J) vs. 6.0±5.46 (Non-J); t (26) = 1.33, *p* = 0.19		

Abbreviations: GAD-7, generalized anxiety disorder (7 items); J, Jewish; PCL, PTSD checklist (20 items); PHQ-9, patient health questionnaire (9 items); PTSD, post-traumatic stress disorder; SD, standard deviation; SSS-8, somatic symptom scale (8 items).

Note: The PHQ-9 item “difficulties of concentration” is missing due to a lack of the online survey; “total burden of complaints” is the sum of the values of the GAD + PHQ + SSS + PCL.

While these findings are exploratory in nature and based on a small, non-representative sample, they should be interpreted with caution and do not allow for causal inference. Beyond descriptive differences, these findings suggest that antisemitism-related stress exposure may operate not only through direct individual experiences but also through broader social environments characterized by heightened vigilance, perceived threat, and uncertainty. The observation that non-Jewish participants also met criteria for clinically relevant symptom burden may reflect indirect exposure pathways, including professional, social, or empathic engagement with antisemitism-related discourse.

Such patterns are consistent with the concept of “stress contagion” within social networks, where exposure to discrimination-related narratives and environments can influence psychological and physiological stress responses even among indirectly affected individuals. This may be particularly relevant in professional settings such as academic or medical environments, where awareness and confrontation with antisemitism may form part of everyday experience.

While these findings are preliminary, they are consistent with the proposed conceptual framework linking chronic psychosocial stress to neuroendocrine and cardiometabolic processes, without allowing causal inferences.

## Mechanistic framework

Chronic psychosocial stress has been proposed as a central biological pathway linking social environments to endocrine and metabolic dysregulation. The hypothalamic–pituitary–adrenal (HPA) axis represents a primary neuroendocrine system mediating this stress interaction, translating perceived social threat into hormonal responses that regulate metabolism, immune function, and cardiovascular activity. Activation of the HPA axis leads to the release of corticotropin-releasing hormone, adrenocorticotropic hormone, and ultimately cortisol. While acute activation is adaptive, chronic activation results in sustained GR exposure, which disrupts homeostatic regulation. 18​

A defining feature of chronic stress is the alteration of circadian cortisol rhythms, including flattening of diurnal variation and impaired negative feedback at the level of GR and mineralocorticoid receptors. These changes contribute to persistent dysregulation of stress-response systems. At the metabolic level, prolonged GR exposure promotes hepatic gluconeogenesis, reduces insulin sensitivity, and alters lipid metabolism. 19​ Cortisol-driven effects on adipose tissue favor visceral fat accumulation, which is associated with increased cardiometabolic risk. Interactions with appetite-regulating hormones such as leptin and ghrelin further contribute to metabolic imbalance. 20​

Chronic stress also affects the autonomic nervous system, leading to sustained sympathetic activation and reduced parasympathetic tone. This contributes to increased cardiovascular load, hypertension, and endothelial dysfunction. Immune dysregulation represents another key pathway. Chronic stress may induce GR resistance, resulting in elevated levels of pro-inflammatory cytokines such as interleukin-6, tumor necrosis factor alpha, and C-reactive protein. This low-grade inflammation is a central mechanism linking stress to cardiovascular and autoimmune diseases. 21​

These interconnected processes are captured by the concept of allostatic load, describing the cumulative physiological burden resulting from repeated stress adaptation. At the systems level, allostatic load reflects the cumulative cost of maintaining physiological stability under conditions of repeated stress. This includes not only endocrine alterations but also structural and functional changes in multiple organ systems. Chronic activation of stress pathways has been associated with vascular remodeling, endothelial dysfunction, and alterations in cardiac autonomic regulation, all of which contribute to the increased cardiovascular risk.

In addition, metabolic consequences of chronic stress extend beyond glucose regulation and include dyslipidemia, altered energy expenditure, and mitochondrial dysfunction. These changes may further interact with inflammatory processes, creating a self-reinforcing cycle of metabolic and immune dysregulation.

Importantly, allostatic load is not solely determined by the intensity of individual stressors but also by their duration, predictability, and contextual meaning, factors that are particularly relevant in the context of chronic and socially embedded forms of discrimination.

However, emerging evidence from related stress paradigms suggests that stress responses are not uniform and may differ substantially depending on individual coping styles and neurobiological adaptations. In our previous work, active, externalizing coping behaviors were associated with lower GR levels despite chronic stress exposure, accompanied by increased striatal glutamate metabolites, indicating a lower allostatic set-point with increased resistance to stress. In contrast, internalizing coping styles are typically associated with higher GR levels and increased vulnerability to stress-related pathology.

These findings highlight the role of fronto-striatal circuits and glutamatergic signaling in modulating stress responses. Increased striatal glutamate activity has been linked to active coping and behavioral engagement, whereas reduced activity is associated with stress susceptibility. The involvement of glutamatergic signaling suggests that chronic stress adaptation extends beyond endocrine regulation to include synaptic plasticity and neural circuit remodeling. Glutamate plays a central role in learning, memory, and behavioral adaptation, particularly within fronto-striatal and limbic circuits. 22​ These mechanisms may also be relevant in the context of chronic antisemitism-related stress, although they have not yet been directly investigated in this specific setting.

Chronic stress has been shown to alter glutamatergic transmission, affecting both excitatory–inhibitory balance and synaptic strength. These changes may underlie differences in coping strategies, behavioral flexibility, and resilience. Increased striatal glutamate activity observed in externalizing individuals may reflect enhanced capacity for active coping and environmental engagement, whereas reduced activity may be associated with behavioral withdrawal and increased vulnerability. Within the present framework, these findings should be understood as a hypothesis-generating extension of the proposed model, suggesting that coping styles and glutamatergic signaling may represent important modulators of stress responses in the context of antisemitism. Future studies may use instruments such as CLASP in combination with neurobiological measures to investigate these interactions more directly.

Beyond cardiovascular and metabolic pathways, accumulating evidence indicates that chronic stress also contributes to cancer initiation and progression through neurobiological mechanisms. Recent advances in cancer neuroscience demonstrate that neural signaling actively shapes tumor biology, including tumor growth, metastasis, and resistance to therapy. Neural activity, mediated in part by stress-related pathways, can directly stimulate cancer cells through neurotransmitter release—particularly glutamatergic signaling—as well as through β-adrenergic and neuroimmune interactions. These processes influence tumor cell proliferation, immune evasion, and metastatic dissemination, positioning the nervous system as a central regulator of cancer pathophysiology. 23​

Importantly, chronic psychosocial stress has been shown to engage these neural circuits, including activation of the HPA axis and sympathetic nervous system, thereby linking sustained social stressors to oncogenic processes. This emerging framework suggests that persistent forms of discrimination-associated stress may not only affect mental and cardiometabolic health but could also contribute to cancer-related biological pathways.

This neurochemical perspective provides a potential link between social experience, behavioral adaptation, and long-term biological outcomes, further supporting the concept of heterogeneous stress-response trajectories. This challenges linear models of chronic stress based solely on hypercortisolism. Instead, it supports a model of differential adaptation in which chronic stress exposure leads to distinct neuroendocrine trajectories, including both hypo- and hyperactivation of the HPA axis.

Within this framework, antisemitism may act as a chronic environmental stressor that interacts with individual neurobiological predispositions, contributing to heterogeneous patterns of endocrine and metabolic adaptation.

## Conclusions

Taken together, the present observations are consistent with a biologically grounded framework in which antisemitism may function as a chronic psychosocial stressor with measurable effects on neuroendocrine regulation and cardiometabolic health. By integrating historical evidence, contemporary exposure, and mechanistic insights, this perspective highlights the importance of considering social environments as biologically relevant determinants of health.

Future research should build on these exploratory observations using larger, well-powered and longitudinal designs as well as combining the psychometric assessment of antisemitic exposure with longitudinal endocrine and metabolic measurements to further elucidate these relationships. It should be noted that the exploratory nature of our pilot data may be subject to selection bias, as participants were recruited through networks with a specific focus on antisemitism, potentially leading to an over-representation of individuals with higher awareness or exposure. Recognizing antisemitism as a health-relevant stressor may contribute to a broader understanding of how persistent forms of social exclusion influence biological systems and disease risk.

In this context, integrating neuroendocrine, metabolic, and neurobiological perspectives may be essential for understanding the full impact of chronic social stressors. Approaches that combine psychometric instruments such as CLASP with biological markers, including endocrine profiles and potentially neuroimaging or metabolic parameters, may provide a more comprehensive assessment of stress-related health effects.

An additional perspective that warrants further investigation concerns the potential psychological and stress-related correlates of individuals expressing antisemitic attitudes. While the present commentary focuses on the health effects of antisemitism on those exposed to it, research into social and personality psychology suggests that persistent negative worldviews, hostility, and perceived threat may themselves be associated with elevated stress levels and adverse health outcomes. In this context, antisemitic attitudes could be conceptualized, in part, as maladaptive coping or projection mechanisms, although empirical evidence on this question remains limited. Future studies may benefit from exploring these bidirectional dynamics to better understand how social hostility and stress may be mutually reinforcing at both individual and societal levels.

Such integrative models may also have implications for clinical practice, as they highlight the need to consider social and environmental factors as part of individualized risk assessment and prevention strategies in cardiometabolic and stress-related disorders.
